# Effect of orthodontic treatment with fixed appliances on the development of gingival recession. A prospective controlled study

**DOI:** 10.1093/ejo/cjaf022

**Published:** 2025-05-28

**Authors:** Dimitrios Kloukos, George Koukos, Nikolaos Pandis, Ioannis Doulis, Andreas Stavropoulos, Christos Katsaros

**Affiliations:** Department of Orthodontics and Dentofacial Orthopedics, School of Dental Medicine, University of Bern, Freiburgstrasse 7, CH-3010 Bern, Switzerland; Department of Orthodontics and Dentofacial Orthopedics, 251 Hellenic Air Force Hospital, Panagioti Kanellopoulou 3, 11525, Athens, Greece; Periodontology, Faculty of Odontology, Malmö University, Carl Gustafs väg 34, 21421, Malmö, Sweden; Department of Periodontology, 251 Hellenic Air Force Hospital, Panagioti Kanellopoulou 3, 11525, Athens, Greece; Department of Orthodontics and Dentofacial Orthopedics, School of Dental Medicine, University of Bern, Freiburgstrasse 7, CH-3010 Bern, Switzerland; Department of Orthodontics and Dentofacial Orthopedics, 251 Hellenic Air Force Hospital, Panagioti Kanellopoulou 3, 11525, Athens, Greece; Periodontology, Faculty of Odontology, Malmö University, Carl Gustafs väg 34, 21421, Malmö, Sweden; Department of Periodontology, Blekinge Hospital, Hälsovägen, Byggnad 13, 371 41, Karlskrona, Sweden; Division of Conservative Dentistry and Periodontology, University Clinic of Dentistry, Medical University of Vienna, Sensengasse 2a, 1090, Vienna, Austria; Department of Periodontology, School of Dental Medicine, University of Bern, Freiburgstrasse 7, CH-3010 Bern, Switzerland; Department of Orthodontics and Dentofacial Orthopedics, School of Dental Medicine, University of Bern, Freiburgstrasse 7, CH-3010 Bern, Switzerland

**Keywords:** gingival recession, orthodontic treatment, fixed appliances, controlled

## Abstract

**Objective:**

To assess in a prospective controlled study whether orthodontic treatment with fixed appliances results in development of gingival recession (GR), compared with an untreated group of participants.

**Materials & Methods:**

The sample consisted of 40 consecutive adult orthodontic patients (Intervention group) and 40 untreated adult volunteers, that satisfied the inclusion and exclusion criteria and were selected from the same background population, as the control group. GR was measured as part of a full periodontal assessment: before treatment (T0) and 12 months after removal of the fixed appliances (T1) in the intervention group, i.e. at about 30 months from T0, and at baseline (T0) and 30 months after (T1) in the control group. A count data model was fit using the sum of recessions at T1 and as predictors: treatment, periodontal phenotype (thin/thick), side (buccal/lingual), sex, age, and number of recessions at baseline, with robust standard errors to account for the multiple within patient observations.

**Results:**

Nineteen females and 21 males in each group [mean age in years (range): intervention group 23.1 (16.8 - 43.3); control: 21.85 (18.2 - 43.9)] were analyzed. During the whole study period, the control group exhibited a modest increase in the number of recessions over time. Several patients in the intervention group exhibited a larger increase in the number of recessions than the controls. However, this was partly counteracted by a considerable amount of reduction in the number of recessions in several patients receiving treatment. The adjusted incidence for recession was 67% higher for the intervention group versus the control group (IRR = 1.67, 95% CIs: 1.05, 2.67, *P* = 0.03). Most recessions, though, were up to 1mm. The most affected teeth were the canines and the first premolars.

**Conclusions:**

Compared to untreated individuals, patients undergoing orthodontic treatment with fixed appliances showed a higher incidence rate of gingival recession at 1-year posttreatment, adjusted for age, periodontal phenotype, side, gender and number of recessions at baseline. However, the severity of gingival recessions was of limited extent.

## Introduction

Occurrence of gingival recession (GR) is affected by various conditions or pathologies [1]; limited width of attached gingiva, gingival thickness of less than 1 mm and a thin alveolar bone have been reported to contribute to recession initiation or enlargement [2–4]. Epidemiological studies have confirmed a high prevalence of GR in the general population; this also seems to increase with age. Over 90% of adults aged 50 years and above were reported to have single or multiple GRs [5, 6]; In another adult U.S. sample aged 30 years or older 22.5% of the participants presented with at least one tooth surfaces with gingival recession of 3 mm or more [7]. The prevalence and extent of recessions was steadily increased with age. In a sample of adults (30-65 years) in France, it was reported that 85% of the participants presented with at least one recession [8]. However, most of the participants (76.9%) had recessions of 1 to 3 mm with severe recessions (of more than 6 mm) found in only 1.8% of the sample. Age, gender, plaque index and tobacco consumption were associated with an increased extent of GR. A recent report on the prevalence of buccal GR, which was based on data from over 10,000 participants, concluded that buccal GR seem to affect almost the entire US population [9]. Female, non-Hispanic white, tooth type (incisors) and mandibular teeth can be considered as risk factors for the presence of GR. However, the results are based mainly on cross-sectional studies, confounding the prospective evaluation of the relationship between age and occurrence of recession.

Although gingival recession has not been linked to increased tooth loss, it often represents an aesthetic problem, it predisposes to tooth hypersensitivity and may hinder oral hygiene. Patients with thin periodontal phenotype are more likely to undergo alteration of the gingival margin during orthodontic treatment irrespective of the type of tooth movement [10]. Previous orthodontic treatment and the presence of malocclusion have been proposed as etiological factors for GR [2, 11]. Orthodontic tooth movement—depending mostly on its direction and the bucco-lingual tissue dimensions—can lead to gingival recession. Prevalence estimates of GR in relation to orthodontic therapy range from 5% to 12% and even up to 47% in the long-term [2]. In a case–control study that followed two samples for almost 9 years, an overall greater odds ratio (OR = 4.48, *P* < 0.001; 95% CI: 2.61–7.70) for recessions in orthodontic cases was reported, as compared to an untreated historical control [12]. Systematic reviews on the topic have indicated that orthodontic therapy was associated with, the clinically insignificant, 0.03 mm of GR (95% CI: 0.01, 0.04) when compared to untreated individuals and that no high-quality evidence regarding the association between orthodontic treatment and the development of gingival recessions is available [13, 14]. On the other hand, orthodontic treatment can help in establishing normal tooth contacts and in positioning teeth optimally within the alveolar envelope, which may, in turn, help to reduce the risk of GR or indeed help to reverse an existing recession. Furthermore, orthodontic tooth movement, with or without orthographic surgery, may improve the apico-coronal tissue dimensions, i.e. coronal migration of the gingival margin, when a facially positioned tooth is moved in a lingual direction [15, 16]. Overall, the major drawback of the studies evaluating recession to date is related to the retrospective study design used.

It seems reasonable to suggest that orthodontic correction of severe malocclusion may help to maintain periodontal health, even in periodontally-compromised dentitions. However, there is no solid scientific evidence on long-term prospective comparisons of untreated cases compared to orthodontically-corrected malocclusions regarding gingival health. Therefore, the objective of the present study was to provide prospective controlled evidence on the effect of orthodontic treatment with fixed appliances on the development of gingival recession.

## Subjects and methods

### Overview of the study design

This study was a single- center, parallel- group, prospective controlled trial. The study included patients starting orthodontic treatment with fixed appliances, followed up for 1-year after completion of orthodontic treatment. An untreated control group, recruited from the same background population, was followed in a similar time frame as the treated group. The control group was recruited on a random basis from two military settings, without previous intraoral examination or other pre-arrangements.

### Trial site

251 General & VA Hellenic Air Force Hospital, Athens, Greece

- Department of Orthodontics and Dentofacial Orthopedics
- Department of Periodontology

### Sample selection and timeline

The sample was obtained from a pool of patients referred to the Department of Orthodontics and Dentofacial Orthopedics, 251 Greek Air Force Hospital, Athens, Greece, for orthodontic treatment needs. Smoking (status and quantity), oral hygiene habits (frequency of toothbrushing/ type of toothbrush), face type, height, weight, and general health status were recorded for possible confounding or etiological link in the development of recession.

Patients were assessed for eligibility according to the following criteria:

Inclusion criteria:

(a) >16 years old
(b) No previous orthodontic or periodontal treatment
(b) Angle Class I up to half cusp Angle Class II dental malocclusion
(c) Space deficit up to 4mm per jaw, without need for extractions
(d) Periodontally healthy as defined by presence of pockets ≤ 3 mm and < 10% of the sites with bleeding on probing (BOP)
(e) No piercing in the lower anterior lip or the tongue
(f) No signs of parafunctions

Exclusion criteria:

(a) presence of crown restorations or fillings involving the cervical part of the anterior mandibular teeth,
(b) pregnant or lactating females,
(c) presence of obvious clinical signs of gingival conditions/diseases resulting in swelling of the gingiva (e.g. gingivitis), or presence of pockets larger than 3 mm at the mandibular central incisors,
(d) presence of labial gingival recessions at the mandibular central incisors,
(e) intake of medication with any known effect on the gingiva, (e.g. Ca antagonists, etc.)
(f) presence of congenital anomalies or dental structural disorders.

The study protocol was approved by the 251 Greek Air Force Hospital ‘Education, Ethics and Research Committee’ (Approval Number: 076/7592/06.05.2015) and was executed in accordance with the guidelines of the Declaration of Helsinki. All patients, or their legal guardian, provided written consent to participate prior to orthodontic treatment commencement or any clinical measurements performed. First patient was enrolled in June 2016 and the last appliance removal took place in May 2020. The last one-year post-debond assessment was performed in May 2021.

Overall, 40 Caucasian patients were included in the intervention group (Group A). Control group (Group B) consisted of 40 healthy untreated individuals matched for age, gender and malocclusion characteristics.

## Description of interventions

### Group A (Intervention)

Group A received fixed orthodontic appliance treatment by one provider (DK) with self-ligating brackets in both arches (In-Ovation R brackets.022’’ slot; Dentsply GAC International, The Hague, Netherlands). Direct orthodontic bonding was done concurrently on both jaws using the Transbond-XT resin material (3M Unitek, Monrovia, California, USA). The archwire sequence was: 0.014” Sentalloy 80 gr (NiTi), 0.016” x 0.022” Neo Sentalloy 80 gr (NiTi), 0.017” x 0.025” stainless steel (SS), 0.019’’ x 0.025’’ SS and 0.017’’ x 0.025’’ Beta-Titanium for finishing, as needed (Dentsply GAC. Islandia, New York, USA). After treatment all patients received a 0.016” x 0.022” stainless steel fixed retainer bonded ribbonwise at all 6 front teeth of both jaws on the day of the appliance removal.

### Group B (Control)

Participants in the control group received no orthodontic treatment or any radiographic evaluation in connection with the study.

## Study assessments

Periodontal examination

The outcomes reported in the present study were evaluated at the following time points:

### Group A:

- Before bracket placement (Baseline- T0)
- One year after Debonding (T1)

### Group B:

- Baseline (T0)
- Thirty months after baseline (T1).

Both groups were clinically evaluated by an experienced periodontist (GK) by means of a fully computerized periodontal probing and charting system, the Florida Probe system (Florida Probe Corporation, Gainesville, FL 32606 USA). This computerized probe system (0.2 mm precision) applies a constant-force and allows measurements to be consistent even when different amounts of force are used during the GR measurements. The possibility of localized gingivitis masking recessions was also recorded. Patients in Group A were given detailed oral hygiene instructions at the day of bonding. In Group B the relevant instructions about oral hygiene procedures were given at their first assessment. Both groups were advised to use of a soft toothbrush and to follow a regular 6-month dental care plan to maintain their oral health status. In every assessment, 168 surfaces were evaluated at all teeth, excluding 3rd molars: 3 on the buccal aspect (disco-buccal, buccal, mesio-buccal) and 3 on the lingual aspect (disto-lingual, lingual, mesio-lingual) at every tooth in every participant. For the categorization of participants as thin or thick phenotype, gingival thickness was measured at all 4 mandibular incisors. Measurements were carried out by the same periodontist (GK) mid-facially on the buccal aspect of each tooth, and 2 mm apically to the free gingival margin, with an Ultrasound device (US); a complete periodontal examination was also performed. Gingival thickness was measured with an US (Krupp SDM®, Austenal Medizintechnik, Cologne, Germany), using the pulse-echo-principle. Measurements were performed by perpendicularly placing the transducer probe on the gingival surface without pressure, ensuring that the center of the transducer would be 2 mm apically to the free gingival margin. The system’s step was set at 0.1mm and 10 consecutive measurements were recorded, and the mean value was registered for each tooth. Based on relevant previous evidence, a participant was categorized as having a thin phenotype when at least one lower incisor had gingival thickness of less than 0.8mm [17].

### Radiographic evaluation (Intervention group only)

Lateral cephalograms have been acquired and Incisor Mandibular Plane Angle (IMPA) was measured at 2 time points:

1. Baseline
2. One month prior to the removal of the appliances. The rationale was to timely detect possible excessive proclination of the incisors. IMPA was calculated as the inner angle formed between the long axis of mandibular central incisor and the mandibular plane [line between gonion and the lowermost point of the mandibular symphysis on the mid-sagittal plane (Menton) (GoMe Line)]. Assessment of intra-observer repeatability and inter-observer reliability in lateral cephalograms was performed using a sample of 15 random chosen radiographs.

### Blinding

All statistical analyses were blinded to the group allocation. Treatment provider and periodontal assessments were not blinded due to the nature of the intervention.

### Method agreement and examiners’ calibration

The validity and examiner calibration of Florida Probe periodontal charting for assessing recessions was performed in 10 randomly selected adult patients by two experienced periodontists. Both performed the full periodontal examination, and this was repeated 2 days later. The kappa statistics was used to assess inter- and intra-observer concordance.

### Sample size calculation

Power analysis and sample size calculation were performed based on the primary outcome: recession in mm. In a relevant study, the gingival recession 5 years post- orthodontic therapy, determined through the mean increase (mm) of clinical crown height of lower incisors, was estimated to be 0.91 mm at the lower incisors’ area with a standard deviation of 0.84 [18]. For such a difference to be detected with 95% power alpha level at 5%, 22 patients per group were required. To account for possible losses to follow up, a total sample size of at least 64 patients (or 32 per group) was calculated.

### Statistical analysis

Descriptive statistics were calculated on the sample characteristics and on the numbers of recessions over time per patient and per tooth. Count data models were fit using the sum of recessions at T1 and as predictors the treatment, side(buccal/lingual), periodontal phenotype (thin/thick), gender, age and number of recessions at baseline with robust standard errors to account for the multiple within patient observations. The model used was Zero-inflated Poisson and confidence Intervals were calculated using non-parametric bootsrapping with 1000 replications. All analyses were conducted using Stata 17 (Stata Corp, TX, USA) and the R Software version 4.0.3 (R Foundation for Statistical Computing, Vienna, Austria).

## Results

### Sample

**Figure 1.**
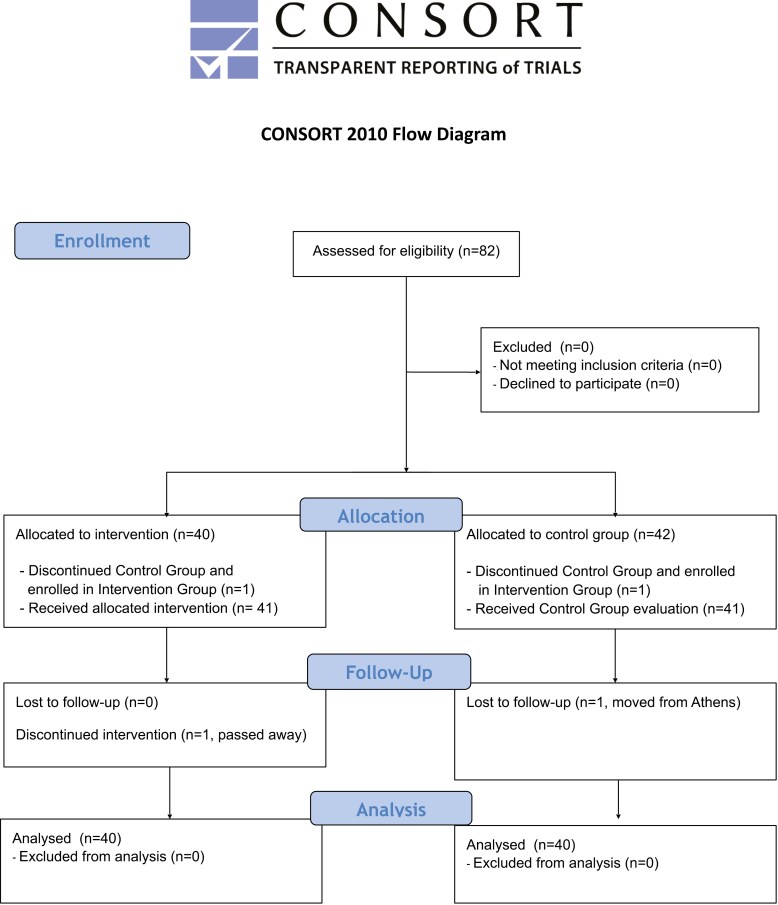
Patients’ flow diagram

Initially, 40 patients were recruited in Group A; one 20-year-old patient passed away unexpectedly during the first year of treatment. Additionally, one patient decided to enroll for orthodontic treatment and moved from Group B to Group A, leaving again 40 participants to be analyzed. In Group B, 42 patients were initially recruited: One patient moved in another country 8 months after study commencement and was not able to attend the follow up appointments. One patient moved to group A for treatment, as mentioned above. In total, 40 patients receiving fixed appliances and 40 untreated participants were analyzed (Fig. 1). Mean age was 23.1 years (range 16.8- 43.3, SD 6.25) in the intervention group and 21.85 years in the control group (range 18.2 - 43.9, SD 7.49). Mean treatment duration until debonding was 21.82 months for the intervention group, i.e. T1 for this group was 33.82 months; mean follow up time for the control group was 30.0 months.

Mean IMPA at baseline in the intervention group A was 92.02^0^ (SD: 5.64^0^). Mean change in IMPA (ΔIMPA) was 6.35^0^ (SD 5.08^0^). For IMPA measurements, intra-observer repeatability was excellent [Intraclass correlation coefficient (ICC) = 0.97] and Inter-observer reliability was good (ICC = 0.82)

### Patient-related risk factors

No participant presented with new piercing on the upper/lower lip or on the tongue after patient recruitment and for the whole study duration. Three patients in the intervention group and 8 patients in the control group were recorded as smokers, with a frequency of 10-20 cigarettes per day. As far as frequency of toothbrushing is concerned, 26 participants in the control group reported brushing once per day and 14 participants brushing twice per day. In the intervention group, 12 patients reported brushing once per day, 24 twice per day and 4 participants three times per day. This indicated a higher frequency of toothbrushing in the intervention group at baseline. Regarding the type of toothbrush, 6 participants in the intervention group and 7 in the control group were using hard toothbrush at study commencement. Thirty-one patients in the intervention group and 24 in the control group were assessed as having thin gingival phenotype.

### Inter- and intra-observer agreement

The kappa scores for the presence of recessions were calculated based on measurements at 6 tooth surfaces of each tooth in every participant. The mean kappa for both inter-and intra-observer agreement for all teeth was larger than 0.85, suggesting almost perfect agreement.

### Treatment outcome (gingival recessions)

**Figure 2.**
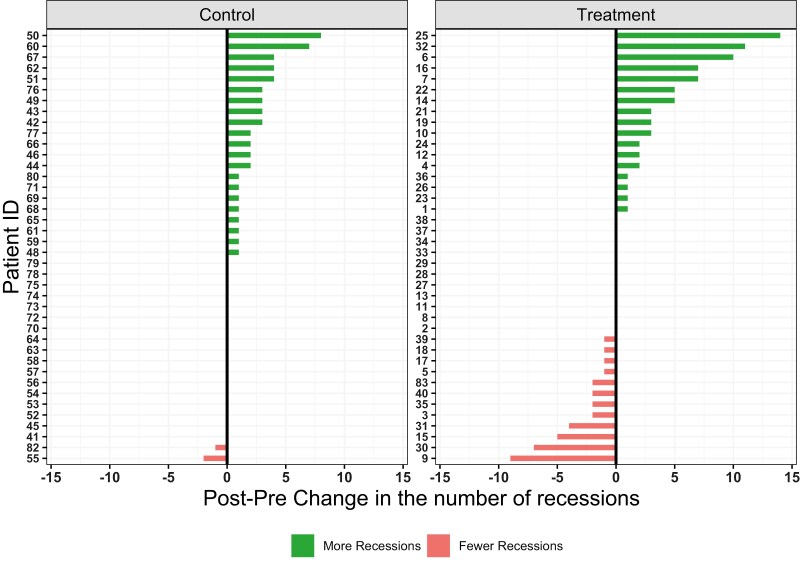
Diverging plots showing the change in the number of GR per patient. Green bars indicate increase and red bars decrease in the number of recessions.

Treatment outcome plots (Diverging and Spaghetti plots, Fig. 2, Suppl. Figure 1) showed variability in the evolution of the number of recessions between baseline (pretreatment) and the final assessment (posttreatment). The diverging plots show in more detail the change in the number of recessions from baseline to the final assessment per group (Fig. 2). Control group exhibited a modest increase in the number of recessions over time. The intervention group exhibited a larger increase in the number of recessions than the controls, although they presented with more recessions at baseline. However, a considerable amount of reduction of recessions was noted in several patients receiving treatment, without presence of gingivitis at any time-point that could have masked GR. All plots depicting number of recessions refer to tooth surfaces presenting recessions out of the 168 surfaces per patient evaluated in every periodontal examination. It should be noted that the recession outcome in mm was converted to binary (0 or 1) since the number of recessions greater than 1mm was small (Table 1). The adjusted incidence rate for the intervention group was 67% higher compared to the control; a statistically significant finding (IRR: 1.67, 95% CIs: 1.05 to 2.67, *P* = 0.03) (Table 2).

**Table 1.** Number of recessions greater than 1mm (per time point and group).

Number of recessions greater than 1mm				
	T0 Intervention	T1 Intervention	T0 Control	T1 Control
**2mm**	3	19	1	2
**3mm**	0	1	0	0

Table 2 shows the results of the Zero-inflated Poisson regression for the Incidence Rate Ratio between groups adjusted for age, periodontal phenotype (thin/thick), side (buccal/lingual), gender and number of recessions at baseline. Except the significance per group, as described before, also males were found to be almost 2 times more susceptible to recession (IRR = 2.04, *P* < 0.02, 95% CIs 1.29 to 3.23). It has to be taken into consideration, though, that confounder effect estimates from a single model are difficult to directly interpret, as these estimates are based on the analysis of the main effect (Table 2).

**Table 2.** Zero-inflated Poisson regression for the Incidence Rate Ratio between groups adjusted for age, periodontal phenotype (thin/thick), side (buccal/lingual), gender and number of recessions at baseline.

	IRR	[95% confidence interval]	*P*-value
**Group**			
** Control**	reference		
** Treatment**	1.67	1.08 to 2.67	0.03
**Age (per unit)**	0.99	0.95 to 1.03	0.53
**Periodontal Phenotype**			
** Thin**	reference		
** Thick**	0.67	0.37 to 1.35	0.27
**Side**			
** Buccal**	reference		
** Lingual**	0.66	0.41 to 1.07	0.09
**Gender**			
** Female**	reference		
** Male**	2.04	1.29 to 3.23	0.002
**Number of recessions at baseline (per unit)**	0.97	0.78 to 1.20	0.75

**Figure 3.**
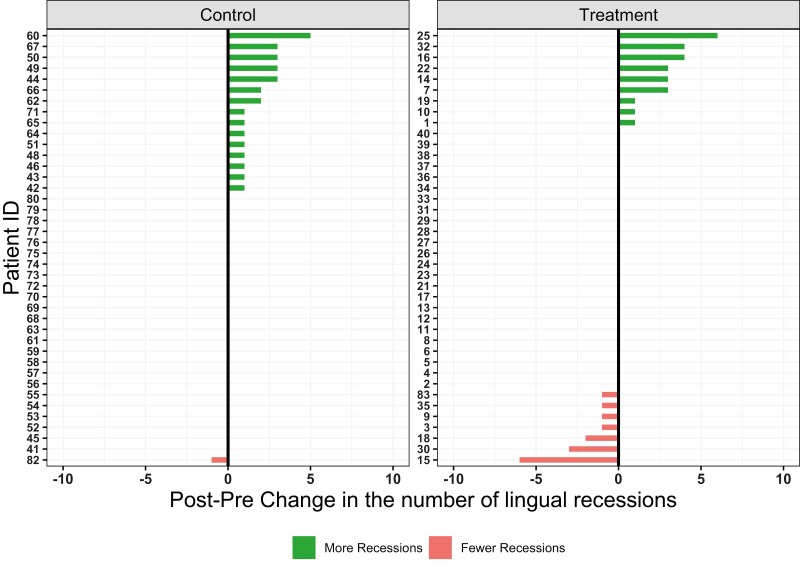
Diverging plots showing the change in the number of lingual recessions per patient. Green bars indicate increase and red bars decrease in the number of recessions.

**Figure 4.**
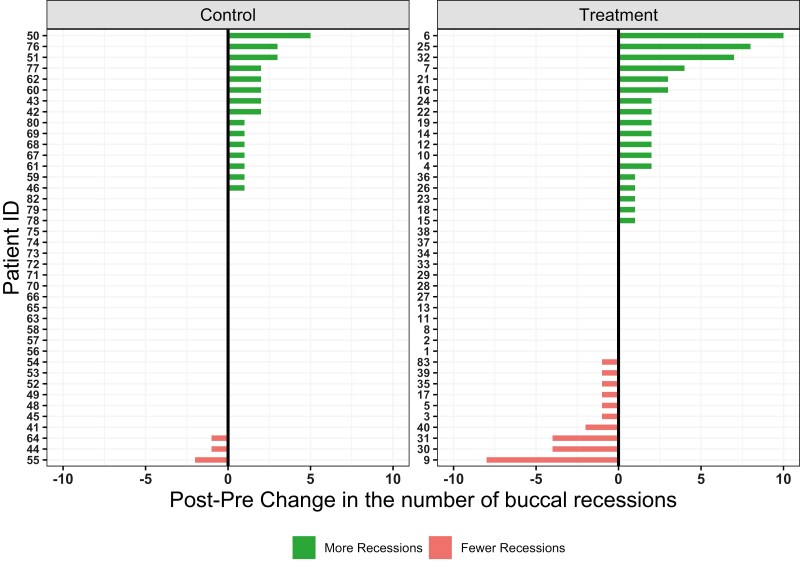
Diverging plots showing the change in the number of buccal recessions per patient. Green bars indicate increase and red bars decrease in the number of recession

When buccal and lingual sides were examined separately, no difference in the shift or trend was noted between groups (Figs 3–4 and Suppl. Figure 2). Again, intervention group followed a similar, but more pronounced pattern in comparison to the control group regarding the number of GR.

**Figure 5.**
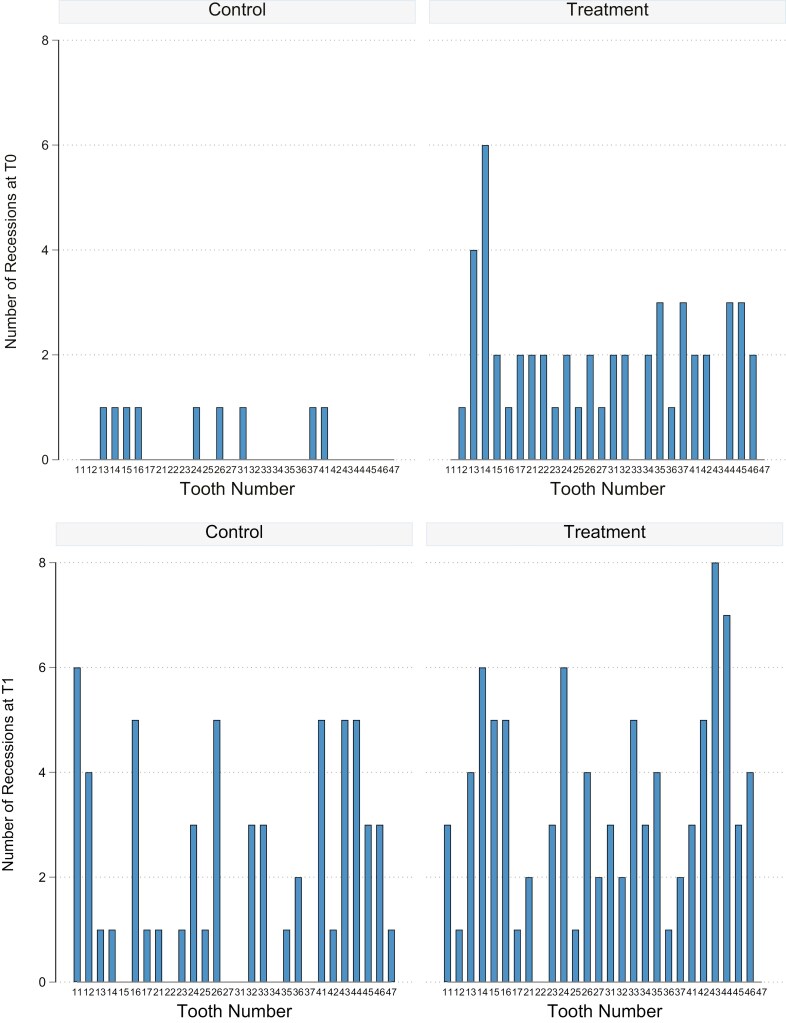
a & b Number of recessions pre- and post-treatment per tooth and group

When only mandibular incisors were analyzed, no clear difference was noted between groups (Suppl. Figure 3). However, data was very thin and statistical analysis could not provide reliable estimates. The predicted number of recessions per treatment and periodontal phenotype was also analyzed, but no statistical difference was reached (Suppl. Figure 4). Figs 5a&b depict the number of recessions pre- and post-treatment per tooth and group.

## Discussion

In the present study, orthodontic treatment promoted the development of gingival recessions. Orthodontically treated patients demonstrated an almost 67% higher incidence rate of recession than untreated controls at 1 year posttreatment. Most recessions were up to 1mm. Nevertheless, clinical significance is often relative in nature; changes in the extent of recession most often do not have any clinical significance in terms of tooth survival, but even subtle changes in the gingival margin may have a great impact in terms of esthetics.

A wide range of general and localized factors have been associated with the development of gingival recession: limited width of attached gingiva, gingival thickness of less than 1 mm, bone dehiscences, high frenal attachment, ectopic tooth eruption, traumatic occlusion, poor oral hygiene and increased accumulation of dental plaque, calculus and smoking via reduced gingival blood flow and destructive oral hygiene habits to remove tobacco staining [5, 19–22]. On the other hand, increased brushing frequency and the use of hard toothbrushes may result in mechanical trauma, which is also reported as a precipitating factor [23–25]. Recession because of traumatic toothbrushing is often encountered on the premolars and the buccal surfaces of teeth, while GR associated with poor oral hygiene is more common in the mandibular incisors and at lingual or proximal tooth surfaces [19, 26–28].

In our study, the Intervention group was found to brush more times per day at baseline, a fact that could partially explain the increased prevalence of recession at study commencement. The use of hard toothbrush was recorded almost equally in both groups and the control group was found to include more smokers that the intervention group, however, the proportion was rather low in both groups. Finally, more individuals with a thin phenotype were recorded in the intervention group, but more GR cannot solely be attributed to this factor, at least at this follow-up. This has probably to do with the statistical analysis; Zero-inflated Poisson regression, among other statistical models, is used to model count data that has an excess of zero counts. Statistical analysis was not easy or straightforward in this study. Although inferences can be given, no clear-cut answer can be given for each contributing factor separately.

Longitudinal measures of the progression of periodontal diseases show that only small changes in periodontal parameters in adults were reported, which may not be directly linked with the natural aging process [29]. However, longer exposure to factors associated with the development of GR may explain the increased prevalence in older individuals. There are localized such as toothbrushing and general intrinsic tissue changes which may have a cumulative effect over time [23, 30]. In the frame of the current study, age did not appear to be a contributing factor. Only gender played a significant role, as males were almost two times more susceptible than females. This is in line with the results of Pernet et al. [31], Albandar and Kingman [7] and Gorman et al. [32]. However, Djeu et al. [33] and Ruf et al. [34] did not find significant differences between the two genders.

In our study the treatment group had overall more recessions at baseline; as individuals with more recessions at study commencement did not exhibit greater recession development throughout the study, the baseline recessions should not be regarded as a patient-related risk factor before initiation of orthodontic treatment. In the current study, the opposite could be supported: orthodontic treatment helped reducing recessions in individuals with recession at baseline. Whether the observed improvements are preserved on the long-term, can only be assessed with a longer follow-up.

As a considerable amount of reduction in number of recessions was noted in several patients, a possible explanation could be that orthodontic treatment assisted in facilitating improved oral hygiene procedures or the tooth movement per se contributed to this direction; it has been reported that the direction of tooth movement and the bucco‐lingual thickness of the gingiva may play an important role in soft tissue alterations during orthodontic treatment [35]. A detailed analysis of the movement of the involved teeth and the analysis of the transversal relations/ canine or group guidance in the occlusion before and after treatment could provide further explanation on this observation.

Our results corroborate the findings of Renkema et al. [12], Allais & Melsen [36] and Slutzkey & Levin [37] that reported up to an overall 4.5 time greater odds for recession in orthodontic cases, as compared to untreated controls. Nevertheless, in all those studies, recessions were assessed on plaster models.

As far as mandibular incisors are concerned, since this is an area of great concern in orthodontics, our results suggest that lower incisors were not particularly affected regarding the development of recession, however, data were thin for firm conclusions. Previous studies have demonstrated that lower incisors are more vulnerable [12, 36]. A change in incisor inclination during treatment has been extensively investigated, but conflicting findings have been reported. Several studies report that proclination per se does not seem to predictably increase the risk of development of gingival recession (GR) in comparison to non-proclined teeth. There are, nevertheless, several opposing reports in the literature [18, 31, 33, 34, 36, 38–46]. In the current study the follow-up (1 year after treatment) was probably too short to confidently evaluate this issue. At the planned 5-year posttreatment evaluation this issue will be revisited.

In the current study an untreated group of participants was recruited and followed longitudinally. In studies evaluating the association of orthodontic treatment and recession so far, existing records from untreated individuals and historical control groups have been used. In the study of Gebistorf et al. [47], the authors evaluated plaster models in both intervention and control groups. Allais & Melsen evaluated recessions through slides in both groups [36]. The study of Slutzkey & Levin, despite the inclusion of controls, cannot be considered as providing controlled evidence [37]. Orthodontic research has attempted to make use of existing longitudinal data from untreated patient cohorts. Both studies of Renkema et al. [12] and Juloski et al. [48] have used as a control group healthy participants drawn from the archives of the Nittedal Growth Material, a longitudinal study conducted by the Department of Orthodontics at the University of Oslo. Renkema et al. assessed plaster models and Juloski et al. plaster models and photos and evaluate recession severity and risk. The treated group derived from university setting in both studies, but, despite the use of the same control group, these studies report conflicting results on the prevalence of recessions [12, 48]. There is largely evidence that the use of historical controls seems to be associated with systematic bias, independently of the intervention group and should therefore be avoided [49]. Overall, the main concerns with records from untreated patients or historical control groups are imbalances in the distribution of patient characteristics, selection bias, information bias and temporal bias. Additionally, the outcomes of such studies pertain mainly to slides and plaster model analysis, which might not be as accurate as detailed clinical examinations.

## Limitations

After calibration and evaluation of intra- and inter-rater agreement, the periodontal assessments were performed by one periodontist (GK). Although possible measurement error would probably be similar between groups, a second periodontal assessment was not ethically accepted or clinically feasible for the whole course of the study. Standardized orthodontic techniques were performed by one orthodontist (DK) with one type of appliance and a certain sequence of wires. This may not be generalizable to other settings/orthodontic procedures. Although thin gingival phenotype was assessed only at mandibular incisors through gingival thickness assessment, we have categorized patients as thin or thick overall for their phenotype. This could be regarded as over-statement, although this has been supported in a previous systematic review [50]. Finally, this study has followed participants for 30-34 months and a longer follow up is needed as the examined conditions evolve slowly over time.

## Conclusions

Compared to untreated individuals, patients undergoing orthodontic treatment with fixed appliances showed a higher incidence rate of gingival recession at 1-year posttreatment, adjusted for age, periodontal phenotype, side, gender and number of recessions at baseline. However, the severity of gingival recessions was of limited extent.

## Supplementary Material

cjaf022_suppl_Supplementary_Figures_1-6

## Data Availability

All data generated or analyzed during this study are included in this article [and/or] its supplementary material files. Further enquiries can be directed to the corresponding author.
