# Supplementary material for: Effect of orthodontic treatment with fixed appliances on the development of gingival recession. A prospective controlled study
Source: Eur J Orthod. 2025 May 28;47(3):cjaf022. doi: 10.1093/ejo/cjaf022 (PMC12116418; doi:10.1093/ejo/cjaf022)

**Supplementary** **Figure 1.** Spaghetti plots showing the summarised evolution of GR per patient and treatment group


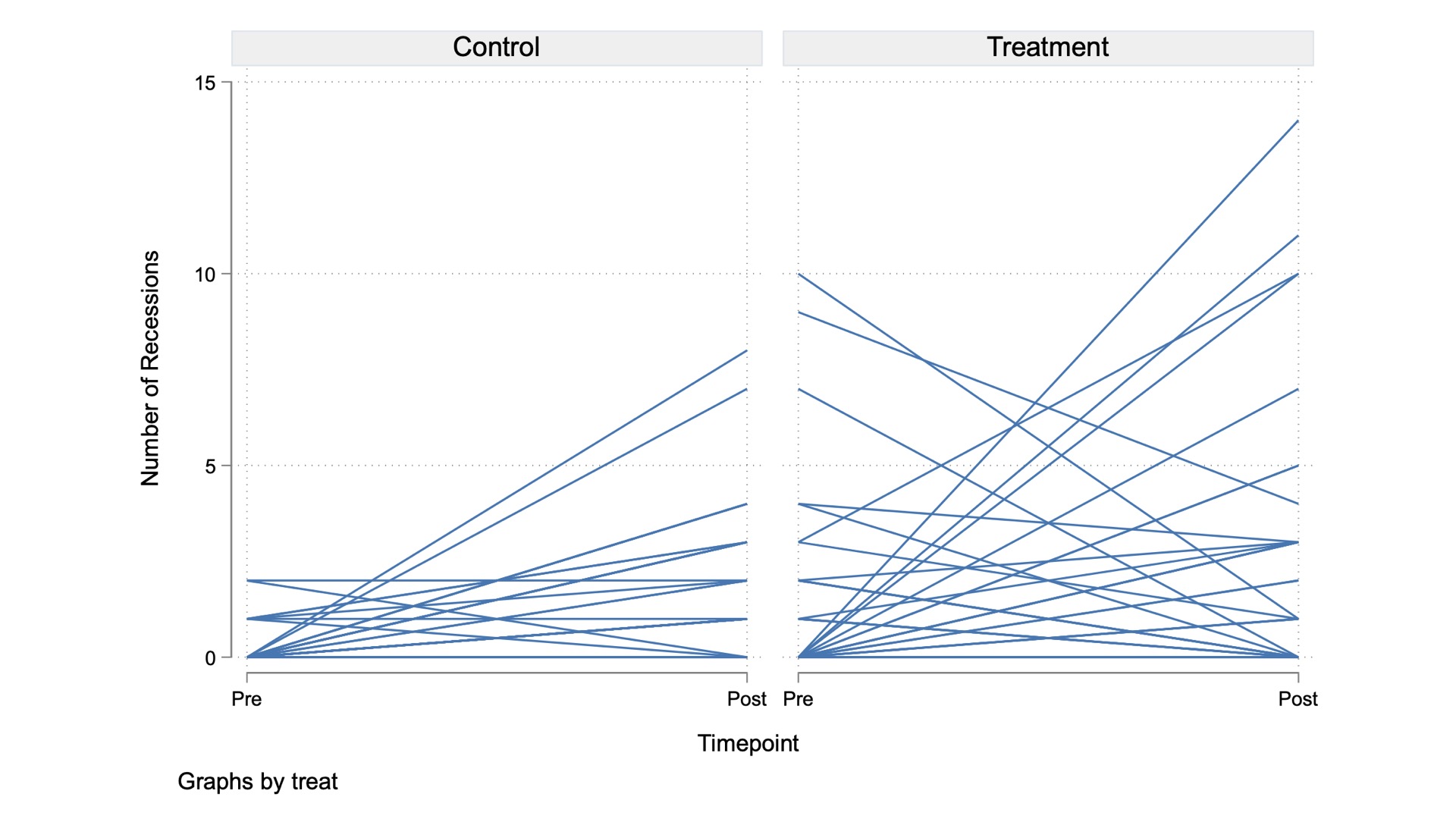


**Supplementary Figure 2.** Spaghetti plots showing the comprehensive evolution of GR per patient and treatment group


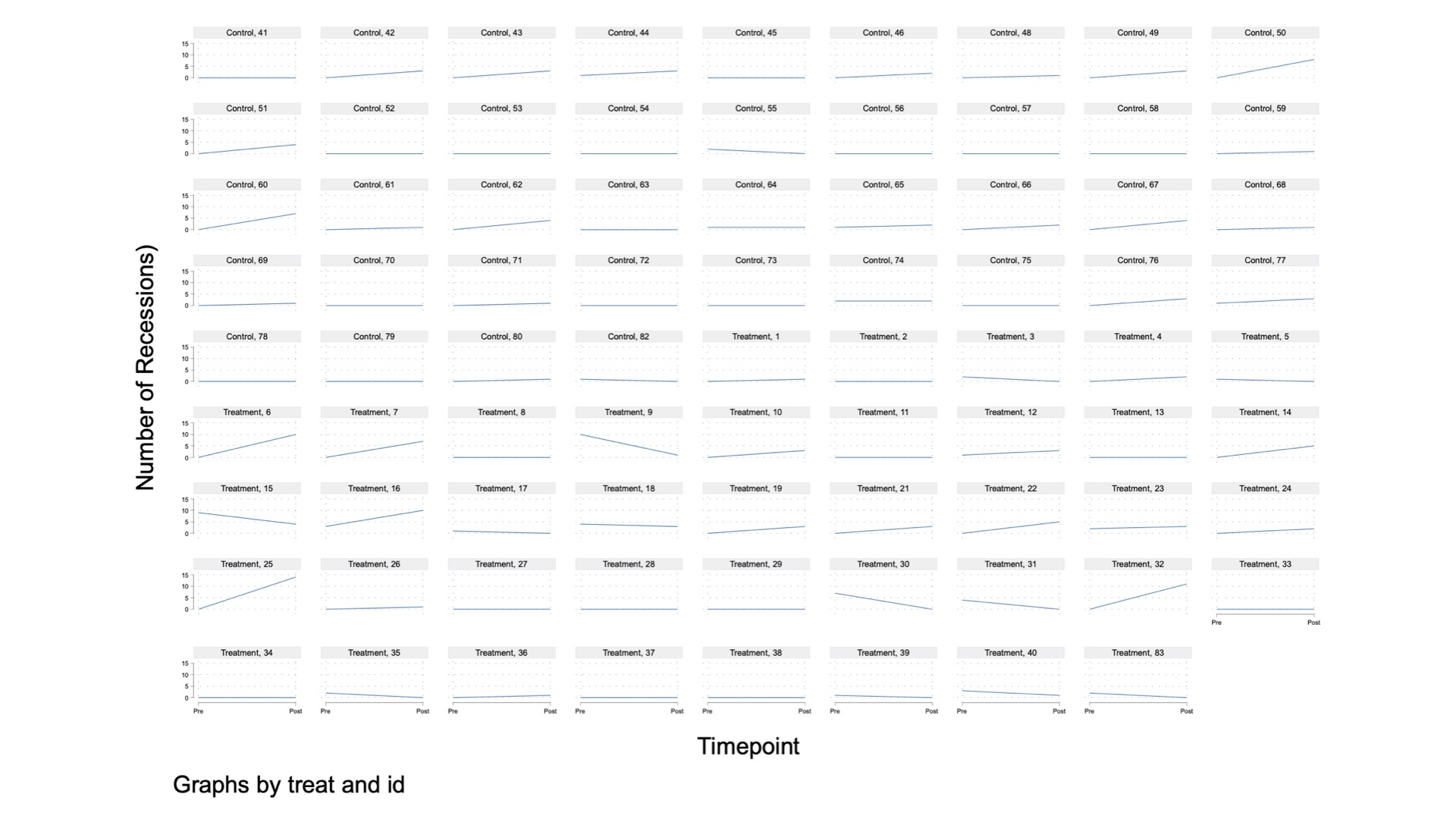


**Supplementary Figure 3.** Spaghetti plots showing the summarised evolution of GR per patient and treatment group per side (buccal/ lingual).


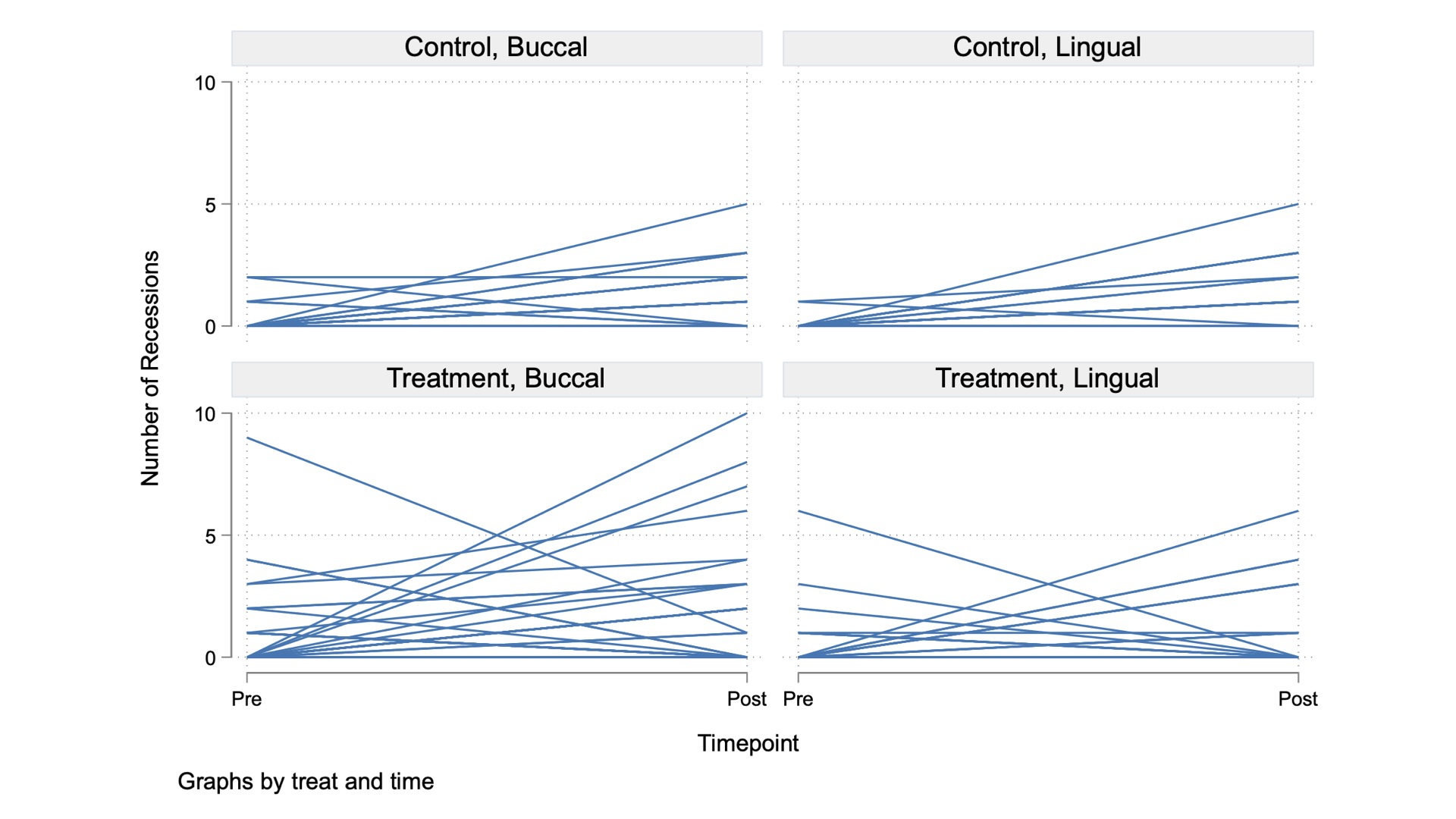


**Supplementary Figure 4.** Spaghetti plots showing the comprehensive evolution of GR per patient and treatment group per side (buccal/ lingual).


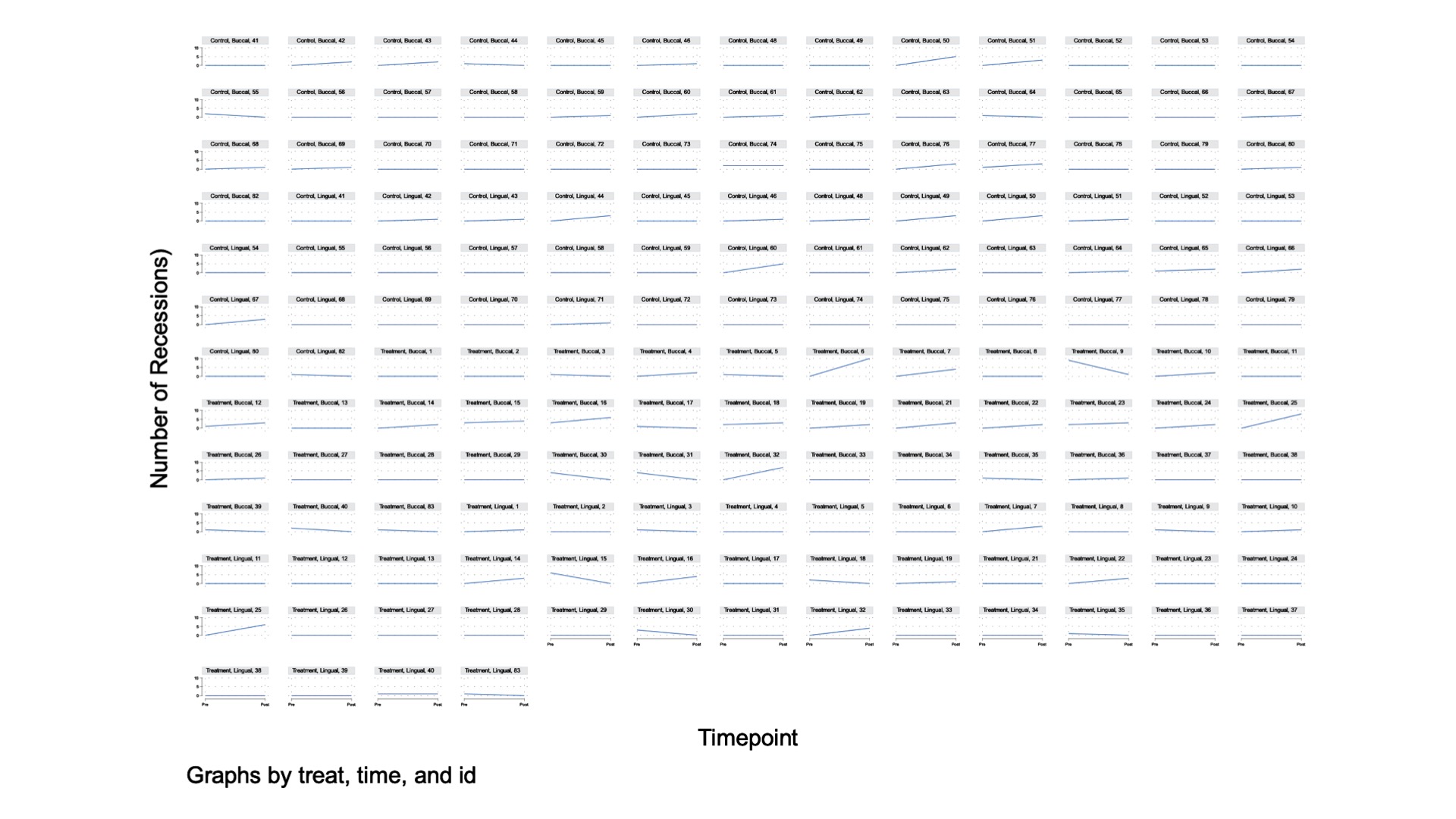


**Supplementary Figure 5.** Spaghetti plots showing the evolution of recessions per patient and treatment group per side (buccal/ lingual) at the mandibular incisors.


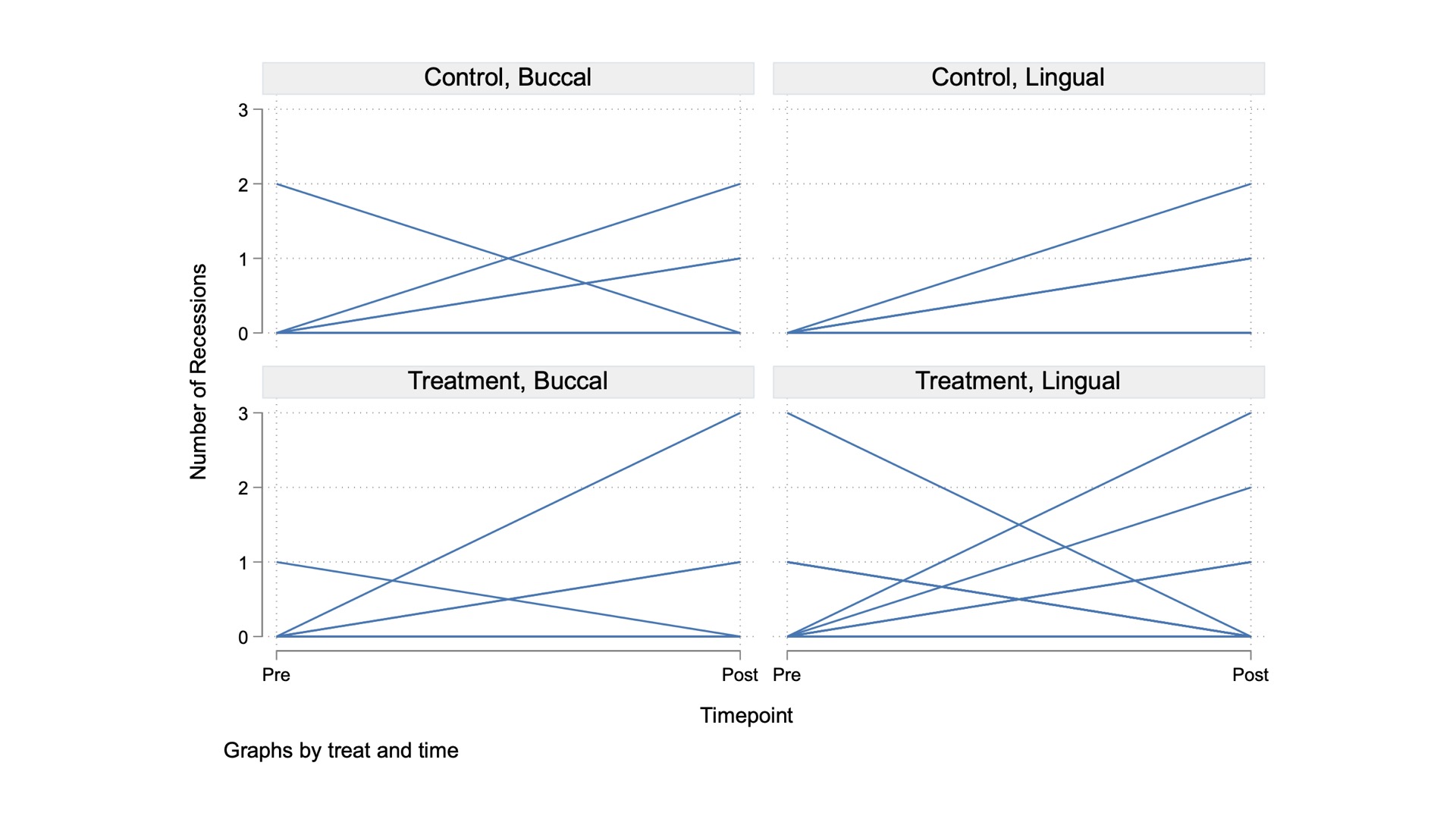


**Supplementary Figure 6.** Predictive margins of treatment events for all teeth


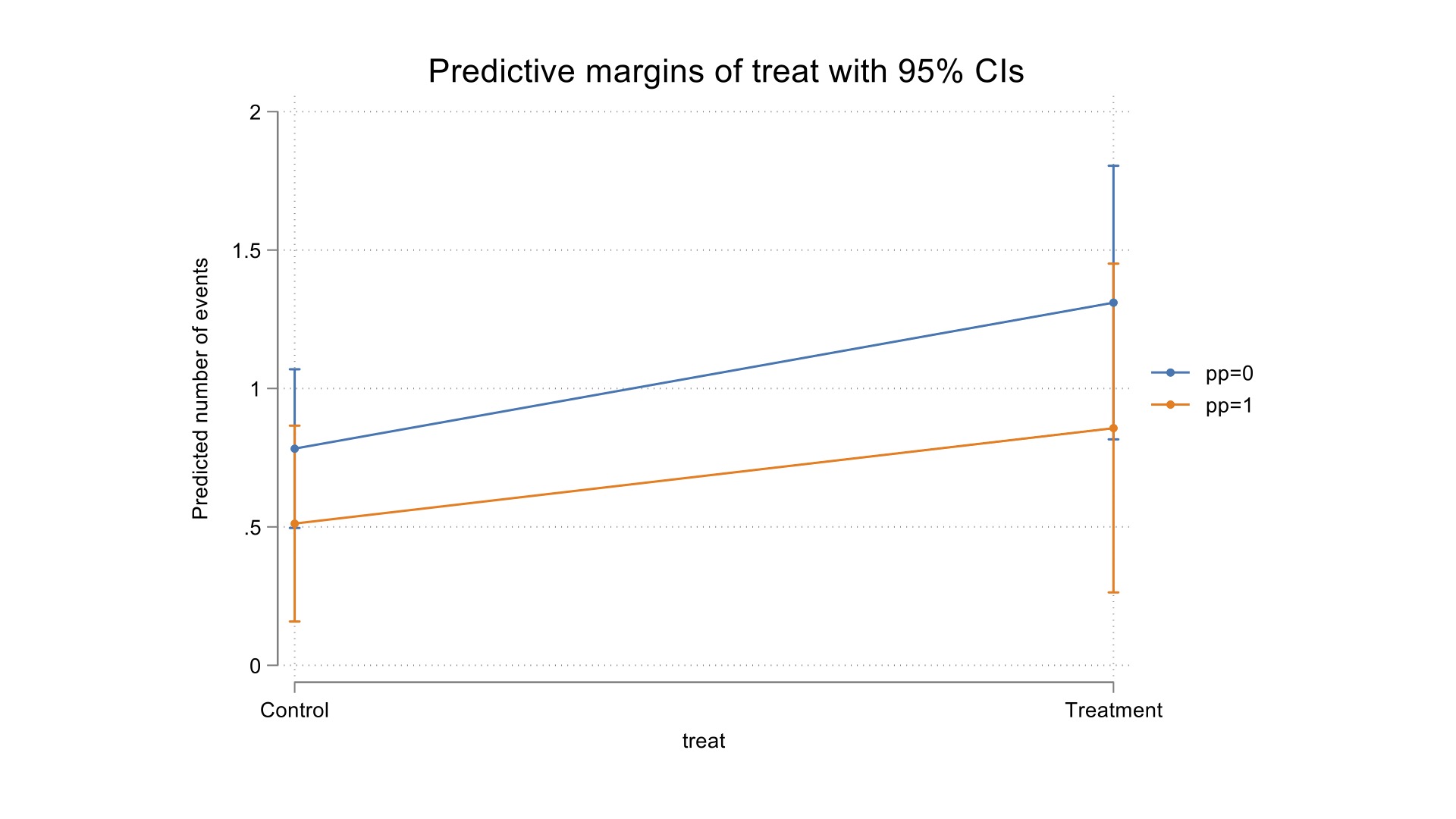

Supplement: cjaf022_suppl_Supplementary_Figures_1-6 [file cjaf022_suppl_supplementary_figures_1-6.docx]
